# Complete mitochondrial genome of the freshwater red alga *Lympha mucosa* (*Rhodophyta*)

**DOI:** 10.1080/23802359.2017.1390417

**Published:** 2017-10-14

**Authors:** Daniel I. Wolf, Joshua R. Evans, Morgan L. Vis

**Affiliations:** Department of Environmental and Plant Biology, Ohio University, Athens, OH, USA

**Keywords:** *Cox1*, Batrachospermales, Nemaliophycidae, streams

## Abstract

We present the complete mitochondrial genome of a newly described freshwater red alga *Lympha mucosa*. The genome was sequenced using the Illumina MiSeq platform. The circular mitochondrial genome is 25,191 bp, contains 46 genes (24 CDS, 20 tRNA, and 2 rRNA), and has an overall GC content of 27.5%. Phylogenetic analyses of the *cox1* gene show the placement of *Lympha mucosa* within the strictly freshwater order Batrachospermales. The four mitochondrial genomes within the subclass Nemaliophycidae sequenced to date are highly conserved in terms of genome size, gene content, and gene synteny.

The red algae are a clade of photosynthetic organisms with most species inhabiting marine environments. However, a small percentage (<3%) is found in freshwater with most of the diversity (∼255 species) within the order Batrachospermales (Sheath and Vis 2015; Guiry and Guiry 2017). To date, only a single mitochondrial genome within this order has been published (Nan et al. 2017). As well, within the broader subclass Nemaliophycidae, only three (*Palmaria palmata*, *Sheathia arcuata* & *Thorea hispida*) of the over 865 species have their mitochondrial genomes sequenced (Yang et al. 2015; Guiry and Guiry 2017; Nan et al. 2017). The *Lympha mucosa* mitochondrial genome adds much needed data to the Batrachospermales and Nemaliophycidae.

*Lympha mucosa* was collected from Kinniconick Creek, Kentucky USA (N 38.496667, W 83.257222) and vouchered in BHO (A-0176). Silica-dried material was ground in liquid N_2_ and genomic DNA extracted using the Nucleospin Plant II Kit (Macherey-Nagel, Düren, Germany). The Illumina MiSeq platform (Illumina Inc., San Diego, CA) was used for two paired-end sequencing runs, one with Illumina MiSeq Reagent Kit version 3 (Illumina, San Diego, CA) (600 cycle) and one with Illumina MiSeq Reagent Kit version 2 (Illumina, San Diego, CA) (300 cycle). Raw reads were trimmed and *de novo* assembled using CLC Genomics Workbench version 10 (QIAGEN, Venlo, Netherlands) (https://www.qiagenbioinformatics.com). The *de novo* assembly produced two contigs (15,209 and 9,445 bp) that were joined as a scaffold and remapped to verify the circular molecule with an average sequencing depth of 102x. Genes were manually annotated in Geneious version 10.1 (Kearse et al. 2012) using Pfam (Finn et al. 2016), Mfannot (http://megasun.bch.umontreal.ca/RNAweasel), tRNAscan-SE version 2.0 (http://lowelab.ucsc.edu/tRNAscan-SE/), and published *Rhodophyta* mitochondrial genomes. Complete mitochondrial genomes of *L. mucosa* and three other Nemaliophycidae (*Palmaria palmata* KF649305, *Sheathia arcuata* KY083064 & *Thorea hispida* KY083066) were aligned using progressive Mauve (Darling et al. 2004) in Geneious version 10.1 (Bio- matters Limited, Auckland, New Zealand). Phylogenetic analyses were performed using the 5-prime region of the mitochondrial *cox*1 gene (COI-5P). Sequences were aligned using MUSCLE (Edgar 2004) and subjected to Bayesian Inference using MrBayes version 3.2 (Software Foundation, Inc, Cambridge, MA; Ronquist et al. 2012) and Maximum likelihood using RAxML version 8.2 (Heidelberg Institute for Theoretical Studies HITS gGmbH, Heidelberg, Germany; Stamatakis 2014) in Geneious plug-ins.

The complete, circular mitochondrial genome is comprised of 25,191 bp and deposited in GenBank (MF488959). It contains 46 genes including 20 tRNA, 24 protein-coding sequences (CDS), and two *rRNA* genes. Overall GC content is 27.5% and total nucleotide content (A, C, G, and T) is 38.5, 13.3, 14.2, and 34.0%, respectively. The phylogenetic analyses showed *Lympha mucosa* within the order Batrachospermales and sister to the genus *Volatus* (Figure 1).

**Figure 1.**
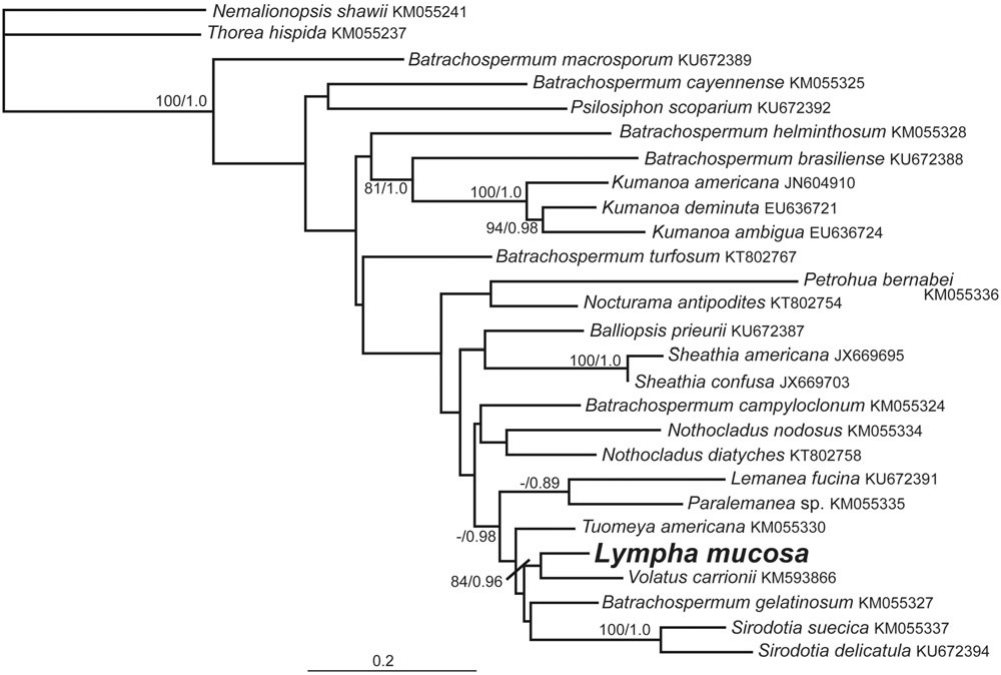
Maximum likelihood phylogeny showing the relationship of *Lympha mucosa* with other taxa of the Batrachospermales and the outgroup species, *Thorea hispida* and *Nemalionopsis shawii* (Thoreales) based on the COI-5P portion of mitochondrial *cox*1 gene. The taxon name and GenBank number are provided. Support values are shown as maximum likelihood bootstrap/Bayesian posterior probability. Branches with no values had support levels <80/0.80.

Whole-genome alignments confirm that the completed Nemaliophycidae mitochondrial genomes are organized into one locally collinear block (LCB) of synteny and are highly conserved. The size of the *Lympha mucosa* mitochondrial genome is comparable to the other freshwater taxa, *Thorea hispida* (25,380 bp) and *Sheathia arcuata* (25,086 bp), but smaller than the marine taxon *Palmaria palmata* (29,735 bp). The CDS among the four Nemaliophycidae genomes are similar, ranging from 23 (*Sheathia arcuata*) to 26 CDS (*Palmaria palmata*). Differences in annotated CDS among taxa were primarily due to open reading frames (ORFs) of unknown function or ORFs with a known function, but incorrectly annotated.
